# Expression Patterns of Sugar Transporter Genes in the Allocation of Assimilates and Abiotic Stress in Lily

**DOI:** 10.3390/ijms23084319

**Published:** 2022-04-13

**Authors:** Zhen Zeng, Tong Lyu, Xin Jia, Yue Chen, Yingmin Lyu

**Affiliations:** 1 Beijing Key Laboratory of Ornamental Plants Germplasm Innovation & Molecular Breeding, China National Engineering Research Center for Floriculture, College of Landscape Architecture, Beijing Forestry University, Beijing 100083, China; zengzhen@bjfu.edu.cn (Z.Z.); jxin95@bjfu.edu.cn (X.J.); chenyue0511@bjfu.edu.cn (Y.C.); 2 Beijing Flower Engineering Technology Research Center, Plant Institute, Management Department of Beijing Botanical Garden, Beijing 100094, China; Lutong123@bjfu.edu.cn

**Keywords:** lily, assimilates, sink-source relationship, sugar transport genes, abiotic stress

## Abstract

During the growth cycle of lilies, assimilates undergo a process of accumulation, consumption and reaccumulation in bulbs and are transported and allocated between aboveground and underground organs and tissues. The sink–source relationship changes with the allocation of assimilates, affecting the vegetative growth and morphological establishment of lilies. In this study, the carbohydrate contents in different tissues of five critical stages during lily development were measured to observe the assimilates allocation. The results showed bulbs acted as the main source to provide energy before the budding stage (S3); after the flowering stage (S4), bulbs began to accumulate assimilates as a sink organ again. During the period when the plant height was 30cm with leaf-spread (S2), leaves mainly accumulated assimilates from bulbs through the symplastic pathway, while when leaves were fully expanded, it transformed to export carbohydrates. At the S4 stage, flowers became a new active sink with assimilates influx. To further understand the allocation of assimilates, 16 genes related to sugar transport and metabolism (*ST* genes) were identified and categorized into different subfamilies based on the phylogenetic analysis, and their protein physicochemical properties were also predicted. Tissue-specific analysis showed that most of the genes were highly expressed in stems and petals, and it was mainly the *MST* (monosaccharide transporter) genes that were obviously expressed in petals during the S4 stage, suggesting that they may be associated with the accumulation of carbohydrates in flowers and thus affect flower development process. *LoSWEET14* (the Sugar will eventually be exported transporters) was significantly correlated with starch in scales and with soluble sugar in leaves. Sugar transporters *LoHXT6* and *LoSUT1* were significantly correlated with soluble sugar and sucrose in leaves, suggesting that these genes may play key roles in the accumulation and transportation of assimilates in lilies. In addition, we analyzed the expression patterns of *ST* genes under different abiotic stresses, and the results showed that all genes were significantly upregulated. This study lays a solid foundation for further research on molecular mechanism of sink–source change and response to abiotic stresses in lilies.

## 1. Introduction

Lilies (*Lilium* spp.) are perennial bulbous plants of *Liliaceae*, with high ornamental and economic value [1]. The growth cycle of lily is unique. During early growth and development, the bulbs serve as the main source organ where starch is the main component of carbohydrates in lilies to provide nutrients for the growth of underground organs and morphogenesis of aboveground organs [2]. As starch is hydrolyzed into soluble sugar, sucrose becomes the main form of soluble sugar that is transported to source organs such as leaves and flowers for both vegetative and reproductive development [3]. While after leaf growth and flower bud formation, with the enhancement of photosynthesis, assimilates produced by aerial parts of plants are transported downward through stems to bulbs and the bulbs gradually transform into sink organs to accumulate assimilates in preparation for the next season [4,5]. Therefore, both the growth and development of the underground organs and the morphogenesis of the aboveground organs are closely related to the allocation of assimilates in lilies, especially starch and sucrose, which play a coordinating and balancing role among various forms of carbohydrate [6].

In higher plants, assimilates produced by photosynthesis are mainly transported long distances in the phloem in the form of sucrose [7]. There are two pathways for sucrose loading and unloading in the phloem: the symplastic pathway is mediated directly by plasmodesmata, and the apoplastic pathway needs the participation of sugar transporters (ST) [8]. After transportation from leaves to fruits, roots and other organ cells, sucrose will be converted into fructose, glucose or Uridine diphosphade glucose (UDP-glucose) by extracellular invertase. Then, glucose and fructose are transformed into glucose-6-phosphate (G6P) and fructose-6-phosphate (F6P) through phosphorylation processes by hexokinase (HK) and fructokinase (FRNK) enzymes, respectively, in order to play roles in live activities, while unmetabolized sucrose is transported into vacuoles via ST [9,10].

The sugar transporters identified in plants can be divided into three types: monosaccharide/polyol transporters (MSTs), sucrose transporters (SUTs or SUCs) and SWEETs (Sugars Will Eventually be Exported Transporter) [11,12]. The MST group is a relatively large gene family, and 53 *MST* genes were identified in *Arabidopsis thaliana* genome, which can be divided into seven clades: STP (Sugar Transport Protein) clade, for which its members played a variety of roles in pollen development and root development [13]; VGT (Vacuolar Glucose Transporter) clade; TMT (Tonoplast Monosaccharide Transporter) clade, PGLUT (Plastidic Glucose Transporter) clade; PLT (Polyol Transporter) clade; INT (Inositol Transporter) clade; and ERD6-like (Early Responsive to Dehydration) clade [14,15,16]. Compared with MST, the distribution range of *SUT* gene family is smaller. Nine *SUT* genes were identified in the *Arabidopsis thaliana* genome, which can be divided into three groups [14,17]. Both MST and SUT belong to major facilitator superfamily (MFS) with 12 transmembrane domains (TMDs) [18]. SWEET, a newly identified sugar transporter, which is different from MST and SUT, contains only seven TMDs and can transport sugars in both directions without energy dependency [19,20]. Currently, the role of *ST* genes in growth and development has been studied in different plants. Sixty-three *ST* genes were searched in grape genome [21], and fifty-two *ST* genes were observed in tomatoes [22]. Thirty sugar transporter and metabolism related genes were identified to play crucial role in development of fruits in peach and apricot [23,24]. *SUT* genes numbering 5, 36 *SWEET* genes in *Petunia axillaris* and 15 *SWEET* genes in *Punica granatum* were found to associated with the flower development [18,25].

In addition to providing the necessary energy for plant growth and development, sugars can also act as signals to regulate plant response to drought, low temperature and other stresses [9,26,27]. *ST* genes can also respond to many forms of abiotic stress. The expressions of *AtSTP13* and *INT*-like gene of *Medicago sativa* were induced by salt stress [28,29], *MdSUT2* could improve the salt and drought resistance in transgenic apple [30]. However, there are a few studies reported to identify the function of *ST* genes in lilies.

The allocation of assimilates in lily is a very complex physiological process affecting sink–source transformation between aboveground and underground organs, which is essential for bulb quality and flower process. Genes related to sugar transport and metabolism may be involved in this process, while little research has been reported about the correlation. Therefore, in this study, carbohydrate contents in different organs at different development stages were determined, and phylogenetic and structural analyses were performed in *ST* genes. Moreover, we conducted a correlation analysis between the expression levels of ST genes and carbohydrate content. Finally, the expression patterns of *ST* genes under abiotic stress were analyzed. These results will provide us new insights for further research on the function of sugar transporter genes in allocation and accumulation of assimilates and abiotic stress resistance in lilies.

## 2. Results

### 2.1. Variation of Carbohydrate Content during Lily Development in Different Tissues 

**Figure 1:**
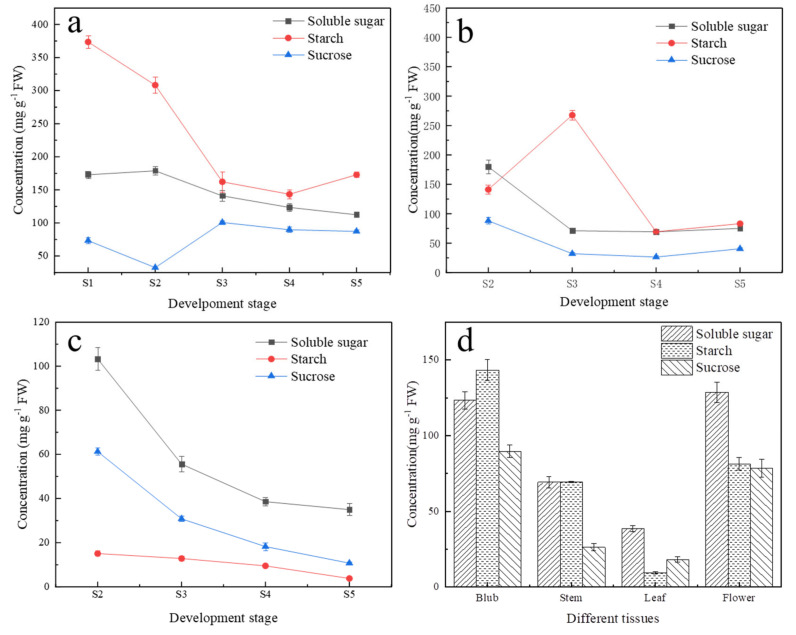
Soluble sugar, starch and sucrose contents (mg/g) in different tissues at different development stages in lilies. Each value indicates Mean ± SE (standard error). (**a**) Carbohydrate contents in bulbs. (**b**) Carbohydrate contents in stems. (**c**) Carbohydrate contents in leaves. (**d**) Carbohydrate contents in different tissues at S4 stage.

The carbohydrate content of different organs in five important stages during lily development was measured, including bulb setting stage (S1), plant height of 30 cm with leaf-spread stage (S2), budding stage (S3), flowering stage (S4) and final flowering stage (S5) (Figure 1). In the scales, the starch content was the highest in S1 stage, and then it continued to decrease from S2 to S4 stage and increased slightly from S4 to S5 stage. The soluble sugar content increased slightly from S1 to S2 stage and then continued to decrease. The sucrose content decreased rapidly at S2 stage and then reached its highest at S3 stage, remaining stable afterward (Figure 1a). In stems, the concentration of starch increased rapidly at S3 stage, which was much higher than that in other periods, and it decreased sharply at S4 stage and then increased slightly at S5 stage. The concentration of sucrose and soluble sugar showed a similar trend, which decreased from S2 to S4 stage and slightly increased at S5 stage (Figure 1b). In leaves, the content of all starch, soluble sugar and sucrose decreased continuously from S2 to S5 stage, and soluble sugar and sucrose decreased rapidly at S3 stage (Figure 1c). These results indicated that from S1 to S3 stage, the bulbs mainly provided energy for plant growth as the source; from the S3 to S4 stage, the carbohydrate content in the aboveground and underground organs was in relatively balance, and the function of bulbs as the source organ was weakened. After S4 stage, the bulbs transformed into the source, reaccumulating assimilates in preparation for the next season. Furthermore, carbohydrate distributions in different organs at flowering stage (S4) were also determined (Figure 1d). In scales, the starch content was the highest, while in leaves and petals, the soluble sugar content was higher than starch, and there was no significant difference in stems. It was also found that, except in scales, most carbohydrates accumulated in the petals during flowering, indicating that the flower became the new sink tissues to input assimilates during this period. In addition, the content variations of sucrose and soluble sugar were basically the same in all tissues and organs, and sucrose content accounted for more than half of soluble sugar under most stages.

### 2.2. Identification and Phylogenetic Analysis of Key Sugar Transporter and Metabolism Related Genes during Allocation of Assimates

**Figure 2:**
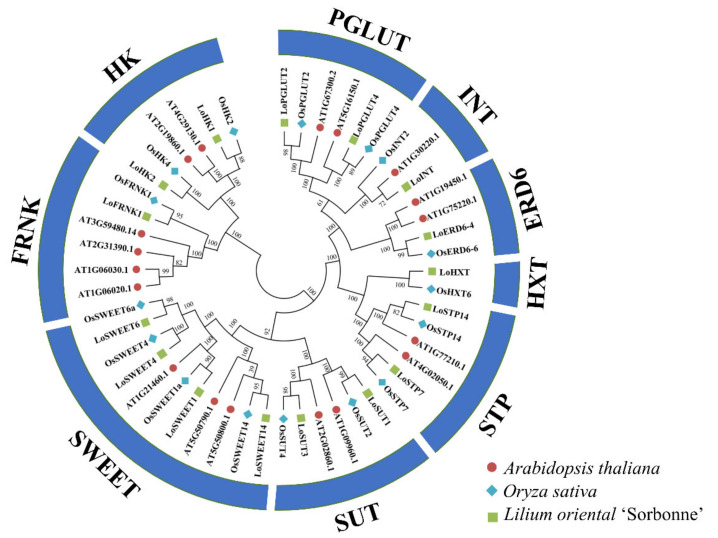
The phylogenetic relationship of *ST* genes in *Arabidopsis* (red), rice (blue) and lily (green).

Based on the protein sequences of *Arabidopsis thaliana* using BLASTp search, 16 genes related to sugar transporter and metabolism were finally identified. A phylogenetic tree consisting of *Arabidopsis* and rice (*Oryza sativa*) ST genes was constructed, which indicated that genes in lilies showed higher similarity with rice, which also belongs to monocotyledons. There are five proteins that belong to the MST family, including a *ERD6* gene, an *INT* gene, a *HXT* gene, two *PGLUT* genes and two *STP* genes; two members belong to the SUT family; four members to the SWEET family; and two forms of kinases including two HK proteins and one FRNK protein (Figure 2). The physicochemical properties of these proteins are shown in Table 1. The coding domain sequences (CDS) of these genes ranged from 633 bp to 1773 bp, and the encoding protein length ranged from 206 bp to 590 bp. The predicted theoretical isoelectric point (*p*I) ranged from 5.21 to 9.41, and the molecular weight ranged from 22.82 kDa to 63.32 kDa, of which the CDS lengths of SWEETs were less than 1000, and the weights of these proteins were the lightest. Ten proteins were predicted to be stable with instability index < 40, and there were significant differences in stability among proteins of the same gene family. With the exception of HK and FRNK, other sequences were transport proteins with transmembrane domains (TMDs) and predicted in plasma membrane localization, of which *LoSWEET*1 were predicted to have five TMDs and LoSWEETS4/6/14 had seven TMDs. LoSTP14, LoPGLUT2 and LoPGLUT4 were predicted to have 10 TMDs, while other MST proteins had 12 TMDs. LoHK1 and LoHK2 were predicted to localized in the chloroplast and LoFRNK1 in the cytoplasm.

### 2.3. Tissue-Specific Expression Pattern of ST Genes 

**Figure 3:**
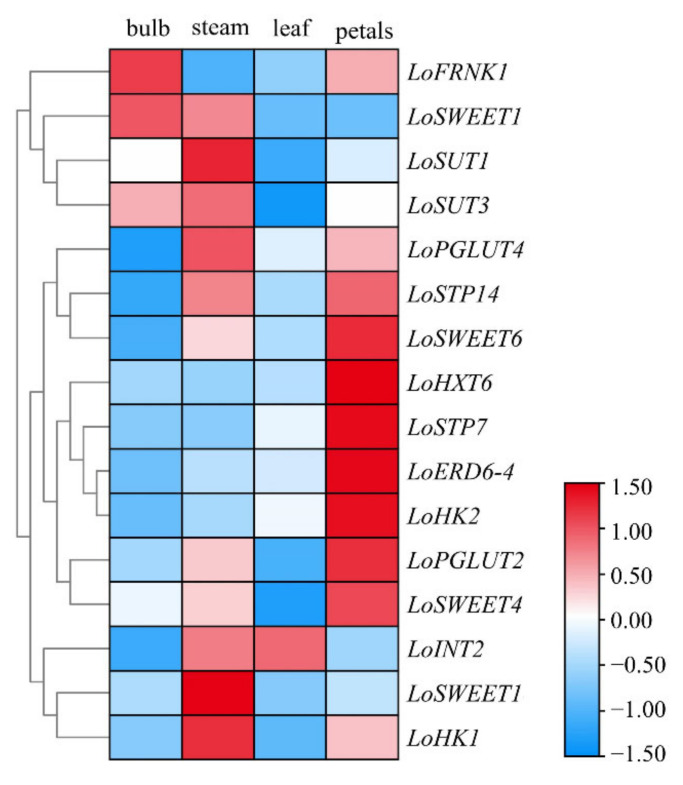
Tissue-specific expression of *ST* genes in lily. The color scale is displayed on the right side, and the color from red to blue indicates the pression level from high to low.

To investigate the transcriptional levels of *ST* genes in different tissues and organs, the scales, stems, leaves and petals of lily at the S4 stage were collected to analyze the expression patterns of these genes by qRT-PCR (Figure 3). Tissue-specific expression analysis showed that all *ST* genes were highly expressed in stems or flowers. Among these genes, *LoSWEET14* was highly expressed only in the stems; *LoHXT6*, *LoERD6-4* and *LoHK2* were only highly expressed in the petals; and other genes were detected in more than one tissues. Only three genes including *LoFRNK1*, *LoSWEET1* and *LoSUT3* showed relatively high expressions in scales, and only *LoINT2* was expressed higher in leaves than in other tissues. *LoHK1*, *LoPGLUT4*, *LoSTP14* and *LoSWEET6* were highly expressed in both stems and petals. *LoSWEET4* was highly expressed in petals, followed by stems, but almost not expressed in leaves. *LoSTP7* was highly expressed in petals, followed by leaves, but almost not expressed in scales and stems. Notably, in petals, all monosaccharide transporter proteins (MSTs) except *LoINT2* were significantly expressed, and *LoFRNK1*, *LoHK1* and *LoHK2* genes were also highly expressed. It is speculated that sucrose may be broken down into monosaccharides such as glucose and fructose to participate in cellular life activities or transported to the vesicles for storage via MSTs. 

### 2.4. Spatial and Temporal Differential Expression Patterns of ST Genes during the Allocation of Assimilates

**Figure 4:**
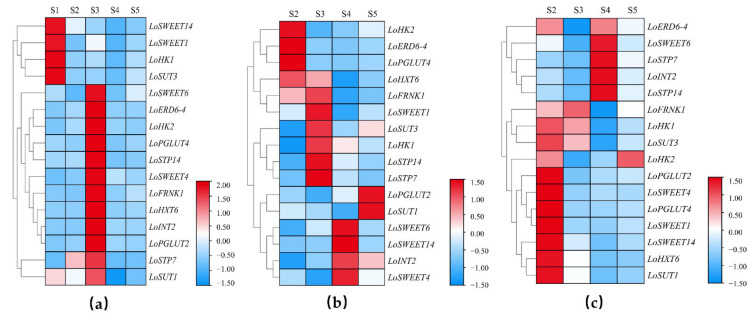
Expression heatmap of *ST* genes during sugar transport in lily in different tissues. (**a**) Expression heatmap in bulb. (**b**) Expression heatmap in stem. (**c**) Expression heatmap in leaf.

To further understand the carbohydrate transport and allocation in development and growth in lilies of underground and aboveground organs, the expression levels of *ST* genes at five stages in scales, stems and leaves were determined and clustered with heatmaps (Figure 4). In scales, most genes were significantly expressed just in a single period. *LoSWEET1/14*, *LoHK1* and *LoSUT3* were highly expressed at S1 stage (Figure 4a). Although carbohydrate transport between aboveground and underground organs had not occurred at this period, starch–sucrose metabolic process still existed to provide energy for maintaining the basic life activities of bulbs, which required the participation of *ST* genes. From S2 stage, bulbs served as the source organ to provide energy for leaf growth, and the contents of starch and sucrose in bulbs began to decline. During this period, the expression levels of *ST* genes did not change significantly except *LoSTP7*, suggesting that sucrose was mainly loaded in the phloem near the source organs (bulbs) via the symplastic pathway, which did not require the mediation of ST. However, in S3 stage, it was also found that the starch content in the scales decreased sharply and a large amount of assimilates flowed out of bulbs, while sucrose content increased significantly (Figure 1a). The expression levels of *ST* genes increased significantly except *Losweet1/14*, *LoHK1* and *LoSUT3*, indicating that, at S3 stage, sucrose loaded in the phloem near the source organs (bulbs) had changed toward the apoplastic pathway, which required the transmembrane transport of ST proteins. The expression levels of *ST* genes in stems, which are necessary channels for the flow of assimilates between aboveground and underground organs, also fluctuated significantly (Figure 4b). The expression levels of *LoHK2*, *LoERD6-4*, *LoPGLUT4* and *LoHXT6* were the highest at S2 stage, while *LoFRNK1*, *LoSWEET1*, *LoSTPs*, *LoHK1* and *LoSUT3* had higher expression levels at S3 stage. *LoSUT1* and *LoPGLUT2* did not change significantly in the early stage but increased suddenly at S5 stage. The expression levels of *LoINT2* and *LoSWEET4/6/14* at S4 stage were significantly higher than those in other stages. The temporal differential expression displayed that the *ST* genes played different roles in the long-distance transport of sucrose in the phloem. In leaves (Figure 4c), most *ST* genes were significantly expressed at S2 stage (except *LoSTPs*, *LoINT2* and *LoSWEET6*) when carbohydrates in bulbs flowed into the not fully mature leaves, indicating that these genes were involved in the accumulation of carbohydrates in leaves as a sink organ. Subsequently, as the leaves gradually matured and photosynthesis was enhanced, the leaves began to serve as source organs to provide energy for lily flowering. *LoSTPs*, *LoINT2* and *LoSWEET6*, which were not actively expressed at S2 stage, increased significantly in terms of expression at S4 stage, revealing their possible involvement in regulating the output of assimilates from leaves to other organs.

**Figure 5:**
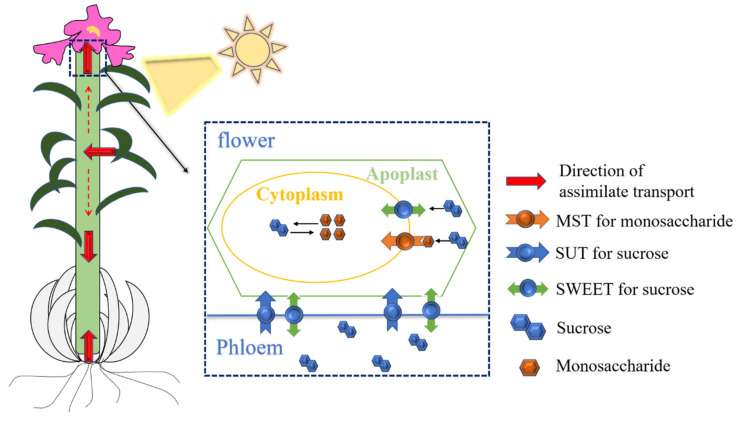
A hypothetical model on the molecular regulation of the allocation and transport of assimilates in lily during flowering.

Based on the results of tissue-specific and spatio-temporal differential expressions of *ST* genes, we proposed a hypothetical molecular regulatory model for assimilating partitioning and transportation in lilies during flowering (Figure 5). The assimilates produced by leaves through photosynthesis flow in large quantities into the petals and also flow downward into bulbs, which are at this point in a dynamic equilibrium of the sink–source relationship. The assimilates are transported in the form of sucrose in phloem and unloaded through the sucrose transporter SUT and SWEET. A portion of sucrose is then transported directly to the cytoplasm via SWEET, while another portion is hydrolyzed to monosaccharides and enters the cytoplasm via MST to participate in vital activities or is stored in the vacuole.

### 2.5. Correlation Analysis between Expression Levels of ST Genes and Carbohydrate Contents 

**Figure 6:**
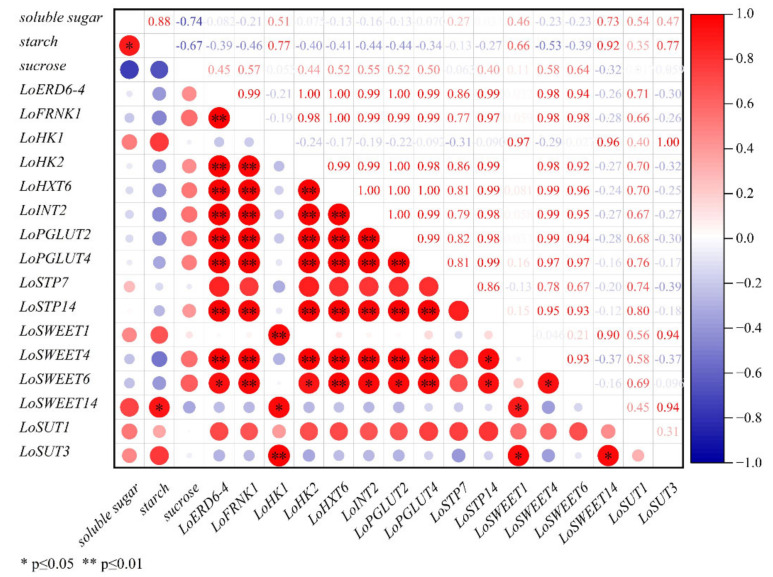
Correlation plot between sugar content and expression levels of *ST* genes during the assimilates transport in bulbs. The figures in the upper triangular indicates correlation values, which determines the size of the circle in corresponding position in the lower triangular. Blue indicates positive correlations while red indicates negative and the color scale is on the right side. * Significant at 0.05 probability level. ** Highly significant at 0.01 probability level.

To obtain a new insight into the relationship between *ST* genes and carbohydrate metabolism, the Pearson’s correlation coefficients in different organs were calculated. In bulbs (Figure 6), the expression levels *LoSWEET14* had significant correlation with starch. There were also significant correlations between the expression levels of sugar transport genes, except for *LoSTP7* and *LoSUT1*. *LoSWEET1/14*, *LoSUT3* and *LoHK1* showed significant correlations with each other, while *LoHK2*, *LoSWEET4/6*, *LoFRNK1* and other *MSTs* showed significant correlations with each other. These findings indicated that they served as both the sink and source during growth and development in lilies, and bulbs played key roles in the accumulation and transformation of photosynthate, which required coordination between multiple genes related to carbohydrate metabolism.

**Figure 7:**
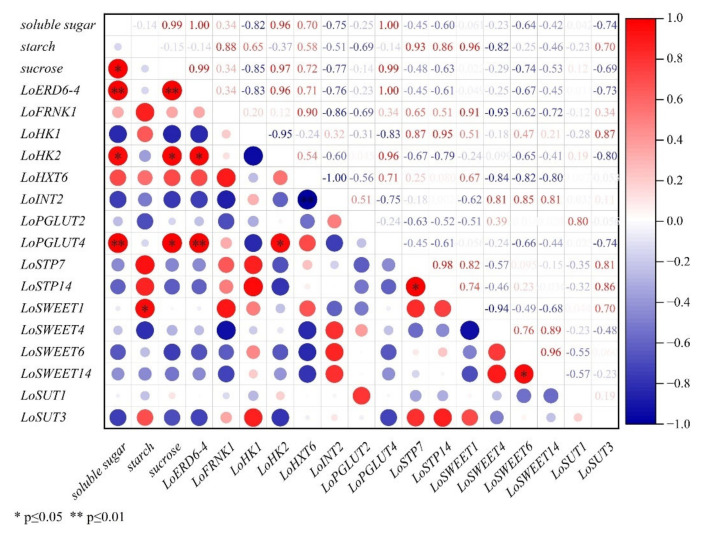
Correlation plot between sugar content and expression levels of *ST* genes during the assimilates transport in stems.

In stems (Figure 7), the expression levels of *LoERD6-4*, *LoHK2*, *LoPGLUT4* showed significant correlations with the concentration of soluble sugar and sucrose. *LoSWEET1* showed highly significant correlation with starch concentration. In addition, the expression levels of *LoHXK2*, *LoPGLUT4* and *LoERD6-4* were significantly correlated. The transcript levels of *LoSWEET6* and *LoSWEET14* were significantly correlated, and *LoSTP7* and *LoSTP14* were also significantly correlated, indicating that these genes may be involved in carbohydrate transport in the phloem. 

**Figure 8:**
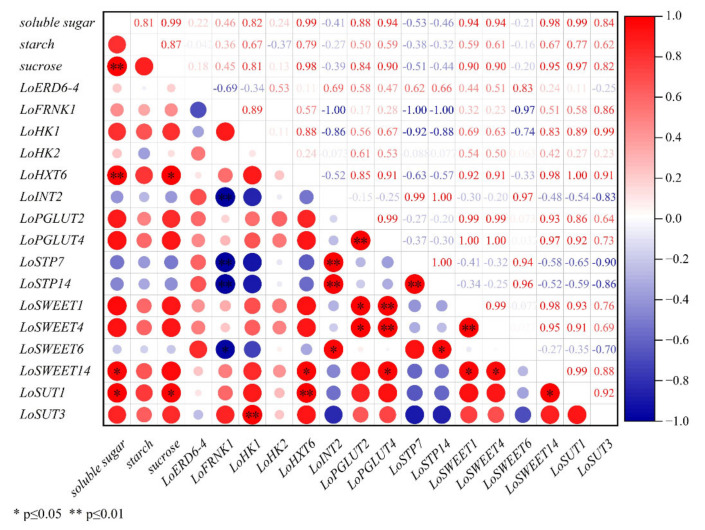
Correlation plot between sugar content and expression levels of *ST* genes during assimilates transport in leaves.

In leaves (Figure 8), expression levels of *LoHXT6* and *LoSUT1* showed significant correlation with the concentration of soluble sugar and sucrose. The transcript level of *LoSWEET14* showed significant correlation with soluble sugar. The expression levels of *LoHXT6*, *LoSWEET14* and *LoSUT1* were significantly correlated. There was also significant correlation in the transcript levels of *LoSWEET1*, *LoSWEET4* and *LoPGLUT2/4*. Moreover, the expression level of *LoSUT3* showed a highly significant correlation with *HK1*. Notably, *LoFRNK1* had significantly negative correlation with *LoSWEET6* and highly significant negatively correlation with *LoINT2* and *LoSTP7/14*.

According to the correlation analysis between the spatio-temporal expression of *ST* genes and carbohydrate content, it was shown that *LoSWEET14*, which was significantly correlated with the accumulation of assimilates in both bulbs and leaves and was also highly expressed in stems, was a key gene involved in the regulation of assimilate allocation and the sink–source relationship of bulbs and leaves. *LoHXT6* and *LoSUT1* also played important roles in the accumulation and transport of assimilates in leaves. In addition, the expression levels of *LoSUT3* and *LoHK1* were highly correlated in both bulbs and leaves, suggesting that they may interact with each other during lily growth and development, which deserves further research.

### 2.6. Eexpression Patterns of ST Genes in Response to Abiotic Stress

**Figure 9:**
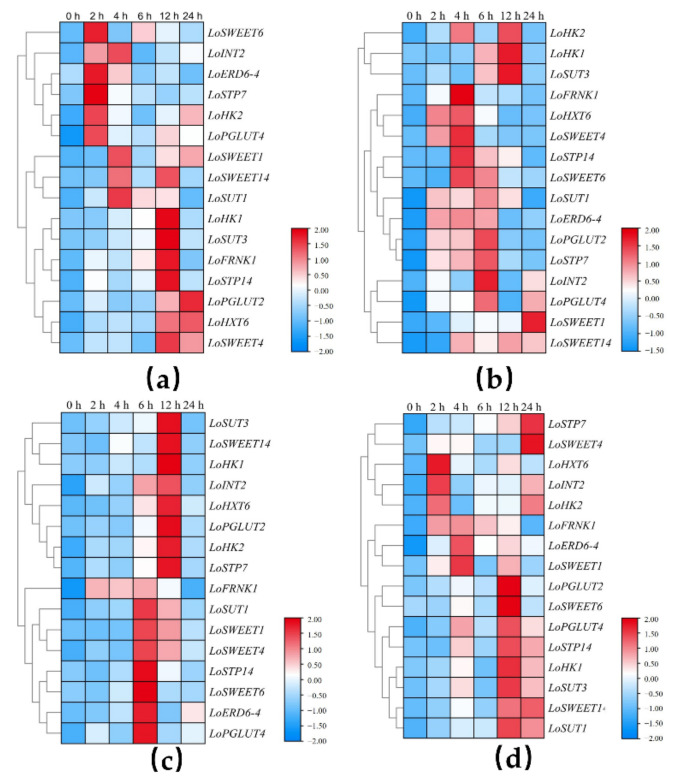
Expression heatmap of *ST* genes after cold (**a**), ABA (**b**), mannitol (**c**) and NaCl (**d**) treatments in leaf.

The expression patterns of *ST* genes under low temperature, drought, salt stress and ABA stress were also analyzed. Compared with the control group (0 h), all *ST* genes showed an upward trend within 24 h after treatment under different stresses. *LoSWEET6*, *LoERD6-4*, *LoSTP7*, *LoPGLUT4*, *LoINT2* and *LoHK2* responded quickly to low temperature, and their expression levels increased rapidly at 2 h while *LoSWEET4*, *LoSUT3*, *LoHK1*, *LoFRNK1*, *LoSTP14*, *LoHXT6* and *LoPGLUT2* showed significant upregulation after 12 h clod treatment (Figure 9a). Under ABA treatment, *LoSWEET1* showed a tendency to accumulate mRNA content with the increase in treatment time. *LoHKs* and *LoSUT3* exhibited a significant increase at 12 h. *LoSWEET14* had a relatively high expression level from 4–24 h after treatment. The expression levels of most genes were significantly upregulated from 2 h to 6 h after treatment (Figure 9b). Under drought stress simulated by mannitol treatments, most *ST* genes showed significant upregulation during 6–12 h, with the exception of *LoFRNK1*, which upregulated significantly at 2 h after treatment and did not decrease until 12 h (Figure 9c). During the salt stress treatment, the gene expression levels of the majority fluctuated wildly. *LoHXT6*, *LoINT2* and *LoHK2* increased significantly at 2 h, then decreased and increased again at 12 h–24 h. The expression levels of *LoERD6-4*, *LoSWEET1/6/14*, *LoPGLUTs*, *LoSTP14*, *LoHK1* and *LoSUT3* upregulated at 4 h, decreased at 6 h and then significantly upregulated again at 12 h. *LoSTP7* increased gradually with treatment times (Figure 9d).

## 3. Discussion

The relationship between carbohydrate metabolism in sink–source organs with plant growth and development has always been a hot research topic [10,31]. During the growth and development of lily, both the reproductive growth of bulbs underground and vegetative growth of flower aboveground are closely related to carbohydrate metabolism [5,6]. In previous studies, the growth and development stages of lily were divided based on the accumulation and consumption of carbohydrates in the bulb, which indicated the role of bulbs in sink–source organ conversion. From bulb setting stage (S1) to budding stage (S3), starch contents in bulb decreased sharply, while soluble sugar and sucrose content did not increase significantly, indicating that the starch was consumed in large quantities after being hydrolyzed. During the leaf-spread stage (S2), the sucrose content decreased significantly in bulbs (Figure 1a), while soluble sugar and starch contents in stems and leaves were the highest (Figure 1b,c). These findings were similar with previous studies that showed that starch stored in bulbs is the main energy source for leaf growth and flower bud development during early stages in which bulbs shouldered the responsibility as a source organ and starch were hydrolyzed into soluble sugar and transported upward to different tissues through the stems. From S3 to S4 stage, with the exception of starch content in stems, the contents of other carbohydrates in different organs did not change obviously. During this period, leaf development was in full swing and could produce more energy through photosynthesis so that carbohydrate consumption and the accumulation of aboveground and underground tissues were in a relatively dynamic balance. During S5 stage, with flowers withered, the carbohydrate content in leaves continued to decrease, while showing an upward trend in stems, and the starch content in bulbs also increased, which indicated that carbohydrates in the aboveground tissues began to transfer to bulbs underground, preparing for growth of the next life cycle. The changes of carbohydrate in different stages in our research were consistent with a previous study on the division of the growth stages of lilies [2,4]. However, studies before were only concentrated on carbohydrate changes in bulbs, while carbohydrate changes of both tissues aboveground and underground were analyzed in our research, which further confirmed previous studies.

In addition, during the entire process of carbohydrate metabolism and transport, the change of sucrose content was consistent with that of soluble sugar, and sucrose content accounted for the majority of soluble sugar, which illustrated that sucrose is the main form carbohydrate transport in lily [32]. It is also worth noting that the soluble sugar content in petals at S4 was the highest among all tissues and organs, and the starch and sucrose contents were also in a high level (Figure 1d). At this time, petals became a new sink tissue, which may be the reason why the starch in the stem increased suddenly in S3 stage and then decreased sharply in S4 stage (Figure 1b), which has not been mentioned before. During floral development in higher plants, with floral initiation or vegetative-to-reproductive transition, a large amount of sugar from photosynthesis was required to flow into floral buds, which became an active sink [33,34]. 

In this study, 16 key genes related to sugar transport and metabolism were identified, and they belonged to nine different gene families including HK, FRNK, INT, HXT, SUT, STP, SWEET, PGLUT and ERD6. All of them were sugar transport, except for HK and FRNK [35,36,37,38,39]. Phylogenetic analysis displayed that most of them had higher homology with rice (Figure 2). Thirteen *ST* genes were predicted to have transmembrane domains (TMDs), among which five to seven TMDs were found in the *SWEET* gene family, and ten to twelve TMDs were found in SUTs and MSTs (Table 1). The SWEETs were predicted to contain seven TMDs and harbored two MtN3 domains in different plant species [16,19], while *LoSWEET1* had only five TMDs. Similar cases were also found in other plants. In watermelon genome, only five TMDs of *ClaSWEET12* were found [40], and the same was observed with *OsSWEET7a* and *OsSWEET7b* in rice [41], and a particular SWEET protein of grape contained 14 TMDs [42]. These SWEET proteins with different TMDs may be caused by domain repetition during evolution, representing the diversity of SWEET protein transmembrane structures [43]. Similarly, the classic structure of MST proteins contains 12 TMDs [22,44], while *LoPGLUT2*, *LoPGLUT4* and *LoSTP14* were predicted to have only 10 TMDs (Table 1). 

The tissue-specific expression analysis of *ST* genes revealed that most of them were highly expressed in stems and petals (especially in petals) (Figure 3), suggesting that these genes may play an important role in carbohydrate transport as well as reproductive development. As mentioned above, during the flowering progress of higher plants, a great amount of sugars flow into the flowers, resulting in a new and active sink organ [33,34]. Sucrose, which is the main form of sugars transported phloem, was broken down to glucose and fructose or stored in cells once imported into flowers [18]. Fructose and glucose can be phosphorylated into glucose-6-phosphate (G6P) and fructose 6-phosphate (F6P) by *FRNK* and *HXK* genes, respectively, and then provide energy for life development [9,10,45]. In this study, we also found that in addition to sugar transporter genes, the transcription levels of *LoFRNK1* and *LoHK2* in petals were relatively high. Studies have been carried out on the relationship between *ST* genes and flower development in some species such as *Arabidopsis*, cucumber and petunia, and it was found that the transformation and metabolism of sugar are influenced by morphological changes and can also regulate flower development in return [18,46,47,48]. However, due to the complexity of flower development and sugar transport, there are few studies on other ornamental plants. In addition, in stems, the change trend of sucrose and soluble sugar was almost the same and the content of sucrose accounted for more than half of soluble sugar content, with all the sucrose transporters (*LoSUT1/3*) and *LoSWEET*s genes showing high expression levels in stems, further indicating that sucrose was the main form sugars transported in phloem [32,49].

Moreover, correlations between expressions of *ST* genes and changes of carbohydrate content during growth and development in lily were performed to identity the most important genes in carbohydrate transporters. In the scales, the expression levels of *LoSWEET14* were significantly correlated with the change of starch content (Figure 6). In the stems, the expression levels of *LoSWEET14* were also significantly correlated with soluble sugar content (Figure 7), and the expression levels of *LoHXT6*, *LoSUT1* showed significant correlations with the concentration of soluble sugar and sucrose, indicating that these genes may play key roles in the accumulation and allocation of carbohydrates to adjust the sink–source relationship. It was also worth noting that Figure 6, Figure 7 and Figure 8 showed there were some correlations among different sugar transporter genes, of which the expression levels of *LoSUT3* and *LoHK1* were highly correlated in both bulbs and leaves, suggesting that they may interact with each other during lily growth and development, which needs to be further researched by using molecular methods.

Otherwise, the function of sugar transporters in response to forms of abiotic stresses has been demonstrated in many plant species [28,50,51]. In addition to responding to abiotic stresses, *ST* genes are also closely associated with plant hormones. In rice, *OsSWEET3a* was not only a glucose transporter but also participated in plant growth through a gibberellin-mediated response [52]. The promoters of *OsSWEET13* and *OsSWEET15* contained ABA responsive elements [53], and the overexpression of *MdSUT2* could improve the tolerance of apple and *Arabidopsis* to ABA stress [30]. In our research study, under low temperatures, salt and drought stress, the transcription levels of MSTs, SWEETs, FRNK and HKs in lilies were significantly changed (Figure 9). Similarly, under the treatment of plant hormone ABA, 16 genes related to sugar metabolism and transport were also significantly upregulated within 24 h of treatment. Based on these results, it is reasonable to speculate that in addition to being involved in sugar transport and distribution, sugar transporter and metabolism-related genes may also play important roles in the regulation network of plant hormones and abiotic stress.

## 4. Materials and Methods

### 4.1. Plant Cultivation and Treatments

The bulbs of oriental lily ‘Sorbonne’ were cleaned, disinfected, dried and stored at 4 °C for 4 weeks before planting. Then box planting was carried out in Beijing Forestry University greenhouse (116.3° E/40.0° N) under the conditions of 70% relative humidity, 25 °C/18 °C day/night temperature and 14 h light and 10 h dark photoperiod. For the determination of carbohydrate content, the samples were obtained at 0 day, 30 days, 48 days, 110 days and 140 days after planting, which were key stages for assimilate transportation in lily, including bulbs setting stage (S1), plant height of 30 cm with leaf-spread stage (S2), budding stage (S3), flowering stage (S4) and final flowering stage (S5), corresponding to the development stage 1–6 described before [4]. The scales of 5 stages, stem and leaves of S2–S5 stages and the petals of S4 stage were sampled. For low temperature stress, the seedlings 8 weeks after planting were cold treated at 4 °C for 24 h. For salt stress, osmotic stress and ABA stress, seedlings were irrigated with 200 mM NaCl, 200 mM mannitol and 150 µM ABA at 8 weeks after planting, respectively. The leaves of seedlings were collected at 0 h (control), 2 h, 4 h, 6 h, 12 h and 24 h immediately. All samples were quick frozen with liquid nitrogen and stored at −80 °C for further analysis, and three biological replicates were performed for each treatment.

### 4.2. Determination of Carbohydrate Content 

The contents of carbohydrate including starch, sucrose and soluble sugar were determined by an improved sulfuric acid-anthrone colorimetric method [54]. Each experiment was performed in three replicates, and data were represented as mean ± SD.

### 4.3. Identification and Phylogenetic Analysis of ST Genes

All FASTAS sequences related to sugar transport in Lilium were identified from the previous transcriptomic data of our laboratory [55,56]. Sequences with full lengths of Open Reading Frame (ORF) were translated into amino acid sequences and compared with diverse plant species by using BLASTp search on the NCBI database (https://blast.ncbi.nlm.nih.gov/Blast.cgi, accessed on 10 January 2022). Only protein sequences with E-value < 1E-3 were preserved for further alignment with ST proteins of *Arabidopsis thaliana* and rice on TRAI (https://www.arabidopsis.org/, accessed on 10 January 2022) and RGAP (http://rice.plantbiology.msu.edu/, accessed on 10 January 2022) databases, respectively. Then, a phylogenetic tree was constructed with MEGA11.0 software according to the NJ method based on protein sequences of lily, *Arabidopsis* and rice. Phobius (https://phobius.sbc.su.se/, accessed on 10 January 2022) and TMHMM2.0 (https://services.healthtech.dtu.dk/service.php?TMHMM-2.0, accessed on 10 January 2022) were utilized to obtain the number of transmembrane domains (TMDs). The ProtParam tools (https://web.expasy.org/protparam/, accessed on 10 January 2022) were utilized to predict molecular weight (MW), theoretical isoelectric point (*p*I) and the instability index of ST proteins. The subcellular localization prediction of each gene was predicated by Plant-mPLoc (http://www.csbio.sjtu.edu.cn/bioinf/plant-multi/, accessed on 10 January 2022) and WoLF PSORT (http://www.genscript.com/psort/wolf_psort.html, accessed on 10 January 2022) [57,58].

### 4.4. RNA Etraction and Quantitative Real-Time PCR

The total RNAs of scales, stems, leaves and petals in different stages and stress treatments were extracted using an EASYspin Plus plant RNA rapid extraction kit (Aidlab Biotech, Beijing, China). RNA degradation and contamination were monitored on 1% agarose gels. First-strand cDNA synthesis was performed using a Prime Script II 1st strand cDNA Synthesis Kit (Takara, Shiga, Japan). qRT-PCR was performed using a Bio-Rad/CFX Connect™ Real-Time PCR Detection System (Bio-Rad, San Diego, CA, USA) with SYBR^®^ qPCR mix (Takara, Shiga, Japan). Three independent biological replicates were used to perform gene expression analyses of all the genes. The relative expression levels were calculated by the 2^−^^△△^Ct method, with *TIP1* as a reference gene [59]. The primers used for qRT-PCR were designed with Primer Premier 5.0 and are shown in Supplementary Table S1. The expression heatmap of all identified *ST* genes was log2 transformed and normalized, and it was constructed by TBtools.

### 4.5. Statistical Analysis

The values of three biological replications were calculated by Mean ± SE. The correlations between expression of *ST* genes and carbohydrate content were measured using Pearson’s correlation coefficient and calculated by using SPSS software. A two-tail test was used to determine the significance level (*p* < 0.05, *p* < 0.01). The correlation plot was constructed using Origin 2021.

## 5. Conclusions

In the present study, the obvious variations of carbohydrate in aboveground and underground organs during the development of lily were observed. Carbohydrates in bulbs underwent the process of accumulation, consumption and reaccumulation so that the role of the bulb transformed between sink and source. The main task of the early underdeveloped leaves was to accumulate assimilates, while when the leaves were fully mature, with the enhancement of photosynthesis, the function of the leaves shifted to source organs, exporting organic matter for plant growth and bloom. During the flowering stage, a large amount of assimilates flowed into the flowers, and the flowers became a new transient sink organ. The expressions of genes related to sugar metabolism and transport changed while assimilates flowed among different tissues and organs. The correlation analysis between carbohydrate content and the expression levels of *ST* genes revealed that *LoSWEET*14 was significantly correlated with starch in bulbs and soluble sugar in leaves, and they were significantly highly expressed in stems, indicating that LoSWEET14 may play an important role in the accumulation of assimilates in lily bulbs and leaves and in the long-distance transport of sucrose in the phloem. In leaves, *LoHXT6* and *LoSUT1* were also significantly associated with soluble sugars, which may influence the process of sink–source relationships in leaves. Furthermore, the expression levels of these *ST* genes increased significantly under various abiotic stress conditions. In conclusion, these results lay a foundation for further research on the role of *ST* genes in regulating the accumulation of assimilates and distribution, altering sink–source relationships and improving resistance to abiotic stress in lilies.

## Figures and Tables

**Table 1:** Characterization of sugar transporter and metabolism genes in lily.

Gene ID	Name	CDS (bp)	Protein Length (aa)	TMDs	*p*I	Instability Index	Molecular Weight (kDa)	Subcellular Localization
Isoform0001530	*LoERD6-4*	1488	495	12	9.15	40.07/N	53.38	PM
Isoform0001773	*LoSUT1*	1495	497	12	9.2	33.71/S	53.17	PM
Isoform0002195	*LoHK2*	1482	493	0	5.76	41.1/N	53.05	Chlo
Isoform0003167	*LoHK1*	1503	500	0	6.34	33.93/S	54.05	Chlo
Isoform0003989	*LoHXT6*	1497	498	12	9.16	38.99/S	54.2	PM
Isoform0008566	*LoSWEET14*	837	278	7	8.49	24.01/S	31.37	PM
Isoform0008671	*LoFRNK1*	999	332	0	5.21	27.58/S	35.82	Cy
Isoform0012368	*LoSWEET4*	777	258	7	9.26	38.49/S	28.64	PM
Isoform0012812	*LoSWEET6*	705	234	7	8.9	40.91/N	25.97	PM
Isoform0015959	*LoSWEET1*	633	206	5	9.41	34.47/S	22.82	PM
Isoform0031035	*LoINT2*	1728	575	12	8.91	40.22/N	62.75	PM
Isoform0031287	*LoSTP7*	1524	507	12	9.37	36.62/S	55.24	PM
Isoform0031586	*LoPGLUT4*	1605	534	10	9.31	35.66/S	56.31	PM
Isoform0031646	*LoSUT3*	1773	590	12	6.86	34.39/S	63.32	PM
Isoform0031860	*LoSTP14*	1533	510	10	8.04	41.18/N	55.96	PM
Isoform0031918	*LoPGLUT2*	1521	506	10	8.29	41.12/N	54.43	PM

CDS: the coding domain sequences; TMDs: transmembrane domains; pI: theoretical isoelectric point; N/S: unstable protein with instability index higher than 40/stable protein with instability index lower than 40; PM: plasma membrane; Chlo: chloroplast; Cy: cytoplasmic.

## Supplementary Materials

The following supporting information can be downloaded at: https://www.mdpi.com/article/10.3390/ijms23084319/s1.

## Abbreviations

The BLAST	Basic local alignment search tool
NCBI	National Center for Biotechnology Information
qRT-PCR	Real-time quantitative reverse transcription polymerase chain reaction
ABA	Abscisic acid
TMDs	Transmembrane domains MFS major facilitator
