# Supplementary material for: Expression Patterns of Sugar Transporter Genes in the Allocation of Assimilates and Abiotic Stress in Lily
Source: Int J Mol Sci. 2022 Apr 13;23(8):4319. doi: 10.3390/ijms23084319 (PMC9029133; doi:10.3390/ijms23084319)
Supplement: Supplementary file 1 [file ijms-23-04319-s001.zip › ijms-1588796-supplementary.pdf]

Table S1 Primers used for qRT-PCR

Gene name	Forward primer sequence (5’- 3’)	Reverse primer sequence (5’- 3’)
LoERD6-4	CGGCATAATCTCCTACACG	CTCCATTGAACAAACAAACCC
LoFRNK1	TGGGATGATCGGGTCGTTTG	GCAGGGATTGCTCCTTTCTTGG
LoHK1	CGTCGTTGCTGCTGTGA	CCCGATTTAGGTAGGAGGC
LoHK2	GCTACTGGGATAATGATGTGATG	TCTCCTGGATTTACGCTTTC
LoHXT6	ACCTATCAGAAATGGCTCTACCG	CAGATGCAAGGGAAACTCG
LoINT2	GGATGGCTCGCTCTTATCGGCTT	GAAGTTCCAATCGCCTCTGT
LoPGLUT2	GTGCCTATGATTGTTGGACCTT	ACTCGCCACCATCCCACGACTTC
LoPGLUT4	CTTGGGAAGTCCTAGCACCCTAT	AGCAACTGCCTTGGCTCTGATT
LoSTP7	CTGTTCTACGCTCCAGTGTTGTT	TGACTCCAAGAATGATAGCCACT
LoSTP14	GCAAGCCCACCTCAACGAAAC	GCTGCCTCTTCTACCATACTTTC
LoSUT1	TTATGGCTCTCTGCTTTGTA	TGTGCGAGTAGAAATCATTG
LoSUT3	TGAGGGAATACTCTTATGGAAC	AGAGGAAATCCGAGAACTGAA
LoSWEET1	CCATCATCATGTACGGCTCAC	CCGGACCATTGTTCTTTCTGT
LoSWEET4	GTGGTTGGCATCATAGGAAAT	TTGAGGAGGGTGGCAAGGTAG
LoSWEET6	GCTCCAGTGCCAACATTCATAAC	CATTCCATAGAAGACCCACAACA
LoSWEET14	TTCAGTTCCGTATGTAGTTGC	AAGCGTTTGGCTCCCTC
TIP1 (reference gene)	GAAGCCAGAAACGGAGAAGAAT	GTAGGGTGGATTGGGAAGA
